# Is the Job Satisfaction Survey a good tool to measure job satisfaction amongst health workers in Nepal? Results of a validation analysis

**DOI:** 10.1186/s12913-016-1558-4

**Published:** 2016-07-27

**Authors:** Neha Batura, Jolene Skordis-Worrall, Rita Thapa, Regina Basnyat, Joanna Morrison

**Affiliations:** 1 University College London, London, UK; 2 Nick Simons Institute, Lalitpur, Nepal

**Keywords:** Job satisfaction, Human resources for health, Tool validation, Nepal

## Abstract

**Background:**

Job satisfaction is an important predictor of an individual’s intention to leave the workplace. It is increasingly being used to consider the retention of health workers in low-income countries. However, the determinants of job satisfaction vary in different contexts, and it is important to use measurement methods that are contextually appropriate. We identified a measurement tool developed by Paul Spector, and used mixed methods to assess its validity and reliability in measuring job satisfaction among maternal and newborn health workers (MNHWs) in government facilities in rural Nepal.

**Methods:**

We administered the tool to 137 MNHWs and collected qualitative data from 78 MNHWs, and district and central level stakeholders to explore definitions of job satisfaction and factors that affected it. We calculated a job satisfaction index for all MNHWs using quantitative data and tested for validity, reliability and sensitivity. We conducted qualitative content analysis and compared the job satisfaction indices with qualitative data.

**Results:**

Results from the internal consistency tests offer encouraging evidence of the validity, reliability and sensitivity of the tool. Overall, the job satisfaction indices reflected the qualitative data. The tool was able to distinguish levels of job satisfaction among MNHWs. However, the work environment and promotion dimensions of the tool did not adequately reflect local conditions. Further, community fit was found to impact job satisfaction but was not captured by the tool. The relatively high incidence of missing responses may suggest that responding to some statements was perceived as risky.

**Conclusion:**

Our findings indicate that the adapted job satisfaction survey was able to measure job satisfaction in Nepal. However, it did not include key contextual factors affecting job satisfaction of MNHWs, and as such may have been less sensitive than a more inclusive measure. The findings suggest that this tool can be used in similar settings and populations, with the addition of statements reflecting the nature of the work environment and structure of the local health system. Qualitative data on job satisfaction should be collected before using the tool in a new context, to highlight any locally relevant dimensions of job satisfaction not already captured in the standard survey.

## Background

The insufficiency of the health workforce is a significant challenge to effective health service provision in most low- and middle-income countries [1]. This insufficiency is commonly related to the number of health workers, the quality of available staff or the distribution of the workforce within a system [1]. Health workers are often concentrated in urban areas and in private practice. However, retention in rural areas and in the public sector is challenged by a range of well-described push and pull factors [2]. Interventions that aim at improving retention of health workers need to be developed, implemented and rigorously evaluated. Job satisfaction among health workers is a good predictor of staff retention [3] and therefore, often used to measure the impact of interventions [4, 5]. Job satisfaction also affects productivity among retained workers [6, 7], and therefore, could be used to measure the impact of vacant posts and absenteeism in rural areas [8].

Several job satisfaction measurement tools have been developed for use in high-income countries [9–12]. These commonly measure an individual’s satisfaction with different aspects of their job, as well as overall job satisfaction [13]. However, they have not been validated for use in low- income countries, where the value of work, interactions between religion and class, and social organisations may be substantially different from those in high- and middle- income settings [1].

One of the most widely used definitions of job satisfaction is “a pleasurable or positive emotional state resulting from the appraisal of one's job or job experiences” [14]. An individual’s satisfaction is derived from a process of comparing an aspect of the current job with a frame of reference [11]. Satisfaction is derived in three possible ways: from differences between what the job offers and what the individual expects; from the degree to which a job fulfills individual needs; and from the degree to which individual values are fulfilled [14]. Some of the factors affecting job satisfaction among health workers are salaries, working conditions, scope for promotion, supervision structures, and the division of work [1, 15–17]). Additionally, the health work force has been facing rising levels of stress due to an increasing disease burden and work force shortage [16]. These factors have a synergistic impact on job satisfaction.

The job satisfaction survey (JSS) was developed by Paul Spector to measure job satisfaction in organisations in human service, public and non-profit sectors [18]. The JSS measures nine dimensions of satisfaction, chosen after reviewing the literature. These dimensions are; pay, promotion, supervision, benefits, contingent rewards, operating procedures, co-workers, nature of work and communication [18]. The JSS asks respondents the extent to which they agree with 36 statements within these nine dimensions [19]. The JSS has been used to measure employee job satisfaction in several high- and middle-income countries including the USA, Singapore, Turkey, Pakistan, Taiwan and Iran [18, 20–24]. As the JSS was developed to measure job satisfaction amongst public sector employees, it is potentially suitable for health service personnel working in government-run facilities. However, given that these contexts are very different from our study area in Nepal, the tool needed to be validated and have its reliability tested in the Nepali context.

### Study setting

Nepal has the second lowest score on the Human Development Index (HDI) among the South Asian Association for Regional Cooperation countries at 0.458. It also has low life expectancy at 68 years. [25]. There are wide and persistent inequalities between rural and urban areas, further compounded by caste discrimination. Those belonging to lower castes (*dalit)* and minority ethnic groups (*janajati*) are poorer and have lower levels of access to political power than higher castes [25].

In Nepal, health services are provided through a hierarchy of tertiary hospitals in urban areas; zonal and district hospitals; and primary health care centres (PHCs), health posts (HPs) and sub-health posts [26]. Districts are divided over geopolitical areas of Village Development Committees (VDCs), and each VDC has a health facility. Quality and access to health services in rural areas is affected by the difficult terrain in many districts, as well as infrastructure, equipment and drug supply issues, and the availability of teams of adequately skilled and supported health workers. Nepal has 0.25 nurses and 0.042 doctors per 1000 population working in public sector health services. This is significantly below the World Health Organisation’s recommended 2.3 health workers per 1000 population. Human resource planning has not kept pace with population growth in Nepal, and there are insufficient public sector positions available in some cadres to meet the needs of the population. There are sufficient health workers being produced and trained, but insufficient posts available for them in the public sector and a glut of health workers in urban areas [27].

Nursing care in rural areas is provided by three cadres of maternal and new born health workers (MNHWs)- upgraded Maternal Child Health Workers (MCHWs), Auxiliary Nurse Midwives (ANMs) and Staff Nurses. MCHWs are local women, who have received 15 weeks training and have been providing counseling and supporting basic service provision in sub- health posts. This post has now been discontinued, and eligible MCHWs are expected to receive ANM training. Only some MCHWs have received this training, but many have been upgraded. ANMs receive an 18 month training course accredited by the Council for Technical Education and Vocational Training, and Staff Nurses study for 3 years to gain a proficiency level certificate in nursing. Sanctioned posts for Staff Nurses only exist at PHC level and above, and these positions are often vacant in rural areas. A survey in 2013 found that only 38 % of Staff Nurse posts in PHCs and 53 % of Staff Nurses posts in district hospitals were filled [28]. Promotion and performance rewards are provided according to the Health Service Act 1997, and are only available to MNHWs employed on permanent contracts. MNHWs continue to play a key role in Nepal’s efforts to increase institutional deliveries and reduce maternal mortality [28–30].

While maternal health indicators have been improving in Nepal, the maternal mortality ratio remains high at 281 per 100,000 live births, and the neonatal mortality rate at 33 per 100 live births [31]. Most women deliver at home [32]. The Government of Nepal has put forward long- and short-term strategies to increase access to maternal health care in rural areas. It is implementing a Skilled Birth Attendant training course to improve the ability of MNHWs to provide safe delivery care in rural areas, encouraging rural health facilities to become birthing centres and provide 24-h delivery services [33]. This encouragement to provide delivery services has not been accompanied by an increase in the number of government posts for MNHWs in birthing centres to provide maternity services. The government is also promoting recruitment of MNHWs on short-term contracts at remote health facilities that are either upgrading to birthing centres, experiencing significant absenteeism among current staff, or have a high number of unfilled posts [34]. The main types of short-term contract MNHWs are those recruited by the district health office (DHO MNHWs) through allocated budgets, and those recruited by the local health facility management committee (HFMC MNHWs) through VDC and health facility funds. Little is known about the effect of this contracting on MNHWs, health systems performance and care provision [35] but there are indications that contracting is improving recruitment and retention of ANMs at the PHC level, where it was reported that 70 % and 75 % of ANM sanctioned posts were filled in PHCs and HPs [28].

This paper reports the findings of a mixed methods study to explore the effect of contracting on MNHWs in western Nepal [36]. The study used a convergent study design, with qualitative and quantitative data collected at the same time point [37]. The aim of the study was to validate established tools for the measurement of job satisfaction and motivation. In this paper, we focus only on validating the survey-tool to measure job satisfaction. It complements another study by Morrison et al. [38] that reports on the validation of a survey-tool to measure motivation in this context.

This paper first describes the sampling process and data collection. We then describe the methods used to validate the tool, and we present our results. Finally, we discuss the findings and recommendations for the use of the JSS to measure job satisfaction among government-employed, facility-based MNHWs in a context such as Nepal.

## Methods

### Sampling and data collection

Our sampling was driven by practical and theoretical concerns. We used a case study design, with the health facility as the case, and sought to compare health facilities and districts with more and less contract MNHWs. Therefore we used data from the Family Health Division of the Department of Health Services to sample two districts with high numbers of contract MNHWs, and one with low numbers of contract MNHWs. We only sampled districts in western Nepal so that we could compare MNHWs on different types of contracts, and compare health facilities in a relatively similar geopolitical environment. One district with high levels of contracting was a remote hill district (Dailekh) with an HDI score of 0.422. The other two districts (Banke and Kailali) were in the plains. Banke has an HDI of 0.475, and Kailali an HDI of 0.467 [39].

We attempted to collect data from all MNHWs present at the time of interview in participating PHCs and HPs that had been providing delivery services for at least 1 year. These facilities have sanctioned posts for ANMs and Staff Nurses. We also sampled sub- HPs that had ANMs or Staff Nurses and had been providing delivery services for at least a year. Across all three districts we collected data in 13 sub-HPs, 29 HPs, 10 PHCs, three District Hospitals and one Zonal Hospital. District and central level stakeholders (12) involved with nursing policy were also purposively sampled. The distribution of sampled institutions across the three districts is summarized in Table 1.

**Table 1:** Distribution of sampled health institutions

District	Total number of eligible institutions (N)	Number of institutions sampled (n)	Percentage of eligible institutions sampled
Dailekh	58	16	28 %
Banke	47	15	32 %
Kailali	44	22	50 %

Data was collected at the health facility in a private room with only the researcher and respondent present. We collected data during the monsoon season and had to adapt our data collection plans because of limited access to some facilities. This has resulted in an imbalance in the sample across the three districts, an acknowledged limitation of the study design.

### Qualitative data collection and management

In Dailekh and Banke districts, semi-structured one-off interviews were conducted with with 78 MNHWs. One ANM in the Zonal hospital in Banke refused to participate because she felt tired of giving interviews to researchers. We asked open-ended questions about what they liked/disliked about their job, what made it easy or difficult to work in that facility, and what would affect their intention to stay or leave. We probed for specific aspects of job satisfaction based on the literature including; community support and utilisation, access to training and leave, colleagues and supervision/management [16, 40]. We also conducted 25 interviews with the health facility in-charges’, 31 focus group discussion (FGDs) with local women and 30 FGDs with the HFMC. One in-charge refused to participate, citing time constraints.. In addition, in each district, semi-structured interviews with the Public Health Nurse (PHN) and the District Health Officer (DO) were also conducted. The objectives of these interviews were two-fold: one, to gain their impressions of how satisfied the MNHWs were; and two, to understand the factors that may affect job satisfaction and retention of MNHWs. The duration of FGDs and interviews was usually between one and two hours.

Two female Nepalese researchers (RT and RB) who were trained and experienced in qualitative techniques were employed to translate the survey from English into Nepali and conduct the pilots. They also collected, entered and translated the qualitative data in the respondents’ home language. Most of the interviews were conducted in Nepali, using Hindi when necessary. In Banke, one interview with the chairperson of the HFMC at a PHC was conducted in Abadhi with a nurse translating to Nepali. Two FGDs with women at PHCs were also conducted in a combination of Abadhi and Hindi and were translated by Female Community Health Volunteers (FCHVs).

Data were digitally recorded, and two additional researchers translated data verbatim into English. Full transcripts were completed for 107 interviews and 46 FGDs. Data from the remaining 15 FGDs and 8 interviews were extracted directly from the audio recordings. Field notes on context and data collection process were also taken and added to the transcripts. Participants did not provide feedback on the findings.

To check the quality of translation, a researcher back-translated three pages of four randomly selected interviews into Nepali and compared these with the recordings. There were few discrepancies. JM observed a sample of quantitative and qualitative data collection in every district (22 MNHWs, 1 PHN, 2 DHO, 3 in-charge, 2 women’s groups, 3 HFMCs). Verbal and written informed consent was obtained from all participants, and the study was granted ethical approval from the Nepal Health Research Council.

### Tool development, quantitative data collection and management

The JSS statements were organised into the following dimensions: Pay/Benefits, Co-workers and Supervisors, Promotion, Work environment and General Job Satisfaction. The JSS was translated and tested on two colleagues not connected with the study, before piloting it on two MNHWs in a nearby health post. One statement “There are *few rewards* for those who work here” was changed to “there are *no rewards* for those who work here” as researchers felt their was little difference in these statements in Nepali and the adapted statement was easier to express in Nepali. No other changes were made to the tool.

The adapted JSS was administered in Nepali to 137 MNHWs in three districts as part of a larger survey that also collected demographic data, employment history and motivation [41]. Data were collected on paper forms and entered into SPSS v20. There were a number of missing responses to the items on the JSS (8 %). Dropping records for those MNHWs with at least one missing response would have significantly reduced the sample to 64 % in a non-random manner, resulting in biased findings. Missing values were therefore imputed using regression modelling [42–44]. All quantitative data were analysed using STATA version 13.

### Validation methodology

Several complementary methods may be used to validate a survey instrument administered in a context different from the one for which it was intended [45]. The Trinitarian ideology advocates the three Cs:

1. Content validity: the extent to which the measure includes the most relevant and important aspects of a concept in the context of a given application;
2. Construct validity: the degree to which scores on a measure relate to other measures, in a manner consistent with theoretically derived *a-priori* hypotheses about the concepts being measured; and
3. Criterion validity: the degree to which the scores of a measure adequately reflect a gold standard.

In this study, we test for content and construct validity. As there is no gold standard for measuring job satisfaction, we do not test for criterion validity. Finally, we also test for reliability and sensitivity as described below.

JM coded the qualitative data from all participants according to the dimensions in the adapted JSS tool, and any additional dimensions that emerged from the data. To consider construct validity and internal consistency, we selected a sub-sample of the qualitative interviews, stratified by whether the respondent appeared to be satisfied or unsatisfied. This sub-sample was selected to saturation, which was achieved within ten qualitative interviews from MNHWs who appeared satisfied with their jobs, and five qualitative interviews with MNHWs who appeared dissatisfied. Finally, job satisfaction scores were compared with the qualitative data to check the reliability of the tool.

The adapted JSS comprises five dimensions of five to nine items each. Respondents were asked whether they strongly agreed, agreed, disagreed or strongly disagreed with each item. The responses were coded to assign a higher value to those who said that they were more satisfied with their jobs and a lower value to those less satisfied. After the missing responses were imputed, a job satisfaction index (JSI) was calculated for each respondent, using principal component analysis.

The first test for content validity was conducted by calculating the degree to which the items in each of the dimensions of the JSS were related to each other. If two items in the same dimension are highly correlated, then one will add little additional information about individual motivation. However, if the items are uncorrelated, they measure different traits or dimensions of motivation. Correlation was measured using the Spearman’s rank correlation coefficient for all items in each dimension. Finally, we explored the missing response patterns to understand the degree of acceptability of questions.

We quantitatively measured the reliability of the JSI by calculating the Cronbach’s alpha (α) coefficient of internal consistency [46, 47]. Cronbach’s alpha assesses the degree to which a set of items measures a single latent dimension. The α values for the JSI and each of its components were calculated and compared against acceptability thresholds [48–51]. We also calculate the theta (θ) coefficient that was developed as a special case of Cronbach’s alpha to specifically account for multidimensionality in an item set [52].

We conducted exploratory descriptive analysis of the data to test for construct validity and sensitivity. Using the JSI, we were able to distinguish between MNHWs who were satisfied with their job and those who were not. We categorised those MNHWs with a JSI less than the median JSI as dissatisfied and those with a JSI higher than the median as satisfied. In addition, we created quartiles of the JSI to distinguish between different degrees of job satisfaction. We then tabulated the average JSI by socio-demographic characteristics of the MNHWs to see how this average score varied, for example, between different MNHWs who belonged to different age groups, ethnicities or salary groups. Table 2 presents an overview of the analytical approaches and data used for establishing the validity of the JSS in Nepal.

**Table 2:** An overview of validation methodology and data used

Type of validity	Data source	Analytical approach
Construct validity	• Qualitative interviews with maternal and newborn health workers (MNHWs) • Quantitative survey with MNHWs	• 10 qualitative interviews with MNHWs who appeared satisfied with their jobs, and five qualitative interviews with MNHWs who appeared dissatisfied. • Comparison of job satisfaction scores with the qualitative data.
Content validity	• Qualitative interviews with MNHWs • Quantitative survey with MNHWs	• 10 qualitative interviews with MNHWs who appeared satisfied with their jobs, and five qualitative interviews with MNHWs who appeared dissatisfied. • Calculating the degree to which the items in each of the dimensions of the Job Satisfaction Survey were related to each other, using the Spearman’s rank correlation
Reliability and internal consistency	• Quantitative survey with MNHWs	Calculating: • Cronbach’s alpha (α) coefficient of internal consistency • Theta (θ) coefficient (special case of Cronbach’s alpha) to specifically account for multidimensionality in an item set
Sensitivity		• Created quartiles of the JSI to distinguish between different degrees of job satisfaction. • Tabulated the average JSI by socio-demographic characteristics of the MNHWs to see how this average score varied by different characteristics.

## Results

### Qualitative validation: definitions and concepts of job satisfaction in Nepal

This section uses the qualitative data to describe how Nepali MNHWs define job satisfaction, and thus considers the content and construct validity of the JSS.

The initial pilots revealed that participants had difficulty understanding and responding to negatively phrased items. Researchers were therefore trained to probe after asking negatively phrased questions to check participant understanding and if necessary, to ask the question again.

#### Content validity

##### Pay and benefits

Many MNHWs commented on their pay and benefits: *“My dissatisfaction is about the money. It is too little to pay for the education of our children. The salary is not enough even to pay for small things.”* (ANM) 1022) *“There is not an adequate return compared to the services we provide in this job, so I think am satisfied, but not fully.”* (ANM 1040) Some MNHWs also complained about their salary not coming on time, causing dissatisfaction: *“the negative part is that we should work 24 h a day and there is a delay in being paid”* (ANM 1005).

Incentives and work-related benefits also affects satisfaction. Incentives included access to training, leave, incentives for overnight working and conducting deliveries, food allowances, health insurance, scholarships for children, hazard allowances, staff quarters and help with furnishings, work uniform allowances, and investment into their provident fund, and pension. One ANM told us how satisfied she was with work-related benefits: “*As we get delivery cases quite often, we receive incentives frequently and that is one advantage. Since I am staying in the staff quarters here, I don’t have to pay rent. There is electricity too*.” (ANM 1009).

##### Co-workers and supervision

Relationships with co-workers and supervisors greatly affected job satisfaction. One Senior ANM explained: “*There are many close friends of mine here…When there are misunderstandings between staff and talking behind people’s backs, then people do not like to work in a place*.” (Senior ANM 2007). Co-workers and supervision also affected satisfaction through links with leave, training, and shift work: “*We co-ordinate. She comes in the morning and I come in the evening, it’s easy as there is no fixed schedule, it’s flexible so it’s easier*.” (ANM 2014).

Absenteeism, unfilled posts, and staff shortages affects how MNHWs feel about their jobs: “*there is a lack of manpower as there are not many staff here. One person has to do everything. For example if one person was looking after family planning and one was looking after antenatal care then the patients would get quality services. Also, when I come to work through the night, the following days’ work is affected*” (Staff Nurse 2030).

Another satisfying aspect of the job for younger MNHWs was the opportunity to learn from colleagues: “*the other ANM sister is also very supportive. She often teaches me*.” (ANM 2013) Inadequate monitoring and evaluation however, was a source of dissatisfaction: “*the district should supervise according to our work. If the work is good then there should be provision of something or they should praise the staff or the facility…there has been no proper evaluation*.” (Senior ANM 2012).

##### Promotion and training

Many MNHWs in our study felt they had no chance for promotion because of their short-term contracts. For their career to progress they needed to study further, or apply for a permanent government job: “*I don’t see any future working as temporary staff in this institution, I have to upgrade my qualifications*” (ANM 1021). Relatively few permanent MNHWs discussed promotion opportunities or the lack thereof.

Training was linked to promotion, with many MNHWs interested in further training. One ANM explained: “*If I had got the chance to study I could have upgraded to Senior ANM, and would have been able to provide better quality services. If I was able to go to the district hospital it would be less remote and I would also get the chance to learn under the supervision of doctors*.” (ANM 1018) Many MNHWs said that increased access to training would affect their job satisfaction: “*if we got training about things we don’t know, that would affect our job satisfaction*.” (ANM 1037).

##### Work environment

Insufficient equipment or inadequate infrastructure appears to negatively affect satisfaction: “*If there were proper arrangements for delivery like if they increased the equipment, and beds. And if there were proper walls constructed, and water was made available then it is possible for me to keep working here.*” (ANM 2022) MNHWs were dissatisfied by a lack of management support for equipment supply: “*They don’t send the delivery sets that we order. It is difficult to manage many patients in a single bed.*” (ANM 2011).

MNHWs who had received skilled birth attendant training particularly noted the lack of equipment, which meant they were unable to manage complications: “*It’s a good place to work if there was equipment, in some things we haven’t been able to apply our skills even though we have received training. I had training 4 years ago, and the vacuum set was made available just the day before yesterday. Now I have forgotten everything. So its very difficult*” (Senior ANM 1033).

##### Community support and job security

Community support and appreciation affect satisfaction: “*the people who deliver here make me feel good because of their good nature…I enjoy it a lot when people here support me and agree with my thoughts.*” (ANM 1036). MNHWs also felt good about communities using available services and being aware of what is good for their health: *“…they have started taking iron tablets and coming for checkups. Now, women directly come here if they have any health problem or difficulties, and that make me feel happy*.” (ANM 1038).

Job security also affects satisfaction. Permanent MNHWs complained about the possibility of being transferred: “*Its difficult to leave this place and go somewhere else and work. You have to arrange the environment (build community support) all over again*.” (ANM 2036). Some contract MNHWs were unsure if their contract would be renewed: “*We are always frightened about our job security… temporary staff are always scared they may lose their job*.” (ANM 1006).

##### Contextual ‘non-work’ factors

The wider literature, and our own research, suggest that work and non-work factors interact to affect satisfaction. Our data show the importance of ‘community fit’, living environment (including access to fresh foods, and good housing), and family support. ‘Community fit’ refers to many aspects of belonging in a community such as a relative who can support them and provide a community connection: “*I am local to this place. I know everybody. I have so many friends and relatives in the community, so I never feel insecure. This would be a problem if I worked in other places*”. (upgraded Maternal Child Health Worker (MCHW) 1019) Being of the same ethnicity, religion or language group as community members also contributed to job satisfaction in the workplace: “*I have my own ethnic community here. I know that other staff can’t make them understand to the extent that I can. (Patients) cannot open up and talk to others like they can talk to me. They talk about all their problems and difficulties with me*.” (Upgraded MCHW 2020).

Some MNHWs also mentioned road access and access to referral centres as factors affecting confidence to conduct deliveries “*We are not able to provide the services that we wanted to…It’s difficult here. We might have a serious bleeding case here but we don’t have any transportation*” (ANM 2011). Sleeping quarters when doing night duties, personal security and distance to home also affected satisfaction: “*I feel secure because my home is not so far from here but for nurses who are not from here, they should be provided with separate rooms.*” (Upgraded MCHW 1013). A supportive family environment enabled MNHWs to work the long hours required of them: “*its difficult to stay here alone…. When a delivery case comes sometimes I don’t even have time to cook for my son…sometimes I have to deal with the delivery case with an empty stomach for 24 h*.” (ANM 1040).

This analysis suggests that the dimensions of the JSS tool concur with the main factors affecting job satisfaction, but that some adaption would improve several dimensions. Statements on equipment, infrastructure and facilities should replace statements about administration. Statements about access to training should replace statements about promotion, along with statements about job security. Finally, statements are needed that capture how MNHWs feel working with the local community.

#### Construct validity

To examine construct validity, we compared the qualitative interview data of 5 dissatisfied MNHWs and 10 satisfied MNHWs with their job satisfaction scores. Self-scoring and qualitative interviews were consistent in 11 out of 15 cases, and selected examples are in Table 3. Four MNHWs appeared satisfied in their qualitative interview, yet had low JSI values. They belonged to the Senior ANM and Staff Nurse cadres (Table 4). These MNHWs were quite dissatisfied with some aspects of their job: for example MNHW 2007 complained about the low salary, lack of equipment, electricity and water, and the lack of appraisal of her work. But she enjoyed working with her colleagues, and was content to live in a community where she was familiar with the culture and had family connections, and she had access to public transport. MNHWs whose scores were inconsistent with qualitative interviews tended to place more emphasis on the positive aspects of their environment, of which few are captured by the JSS. Because JSI values and qualitative data were usually consistent, we can say that the tool has an acceptable level of construct validity, but would be further strengthened by adding a social environmental dimension.

**Table 3:** Comparing qualitative and quantitative job satisfaction data

ID number	Job Satisfaction Index (JSI) (quartile category)	Interpretation of qualitative interview	Maternal and Newborn Health Worker (MNHW) interview quotes
1013	4.65 most satisfied	Satisfied	“Community members and my relatives … recognize me as a good person when I support them in their health related problems and during pregnancy and delivery. I really love that.” “There are no problems as the equipment are enough ” “I feel satisfied because I feel capable of doing things like immunization, conducting outreach clinics, giving antenatal and postnatal check-ups, assisting deliveries, managing medicine etc.”
1026	2.31 most satisfied	Satisfied	“But here, because of transportation facility, it is easier to go from one place to another. Because of availability of facilities here, it is difficult for me to leave this place.” “Compared to other places, [the community] is quite supportive. They come to the health facility and are friendly with the health workers and they are interested in getting information.”
2023	2.12 more satisfied	Satisfied	“I feel satisfied because this is my place and my office is near my house. When I am on duty for 24 h, it is easy to go home and then my friends inform me if there is a case.” “Everything is good. All my friends, sisters and sirs are cooperative and friendly. They teach me the things that I don't know. So everything is nice.”
1040	−1.69 less satisfied	Dissatisfied	“The HMC said that they would increase my salary after 1 year, but it has been 4 years already and nobody cares about it.” “Because there is a shortage of drinking water – it only comes every 10–15 days - I don’t feel like staying here. Sometimes I feel sad living alone here and being responsible for everything all by myself.” “We keep on talking (about the lack of rooms), time and time again to our in-charge…he says that it will be done, but nothing has been done so far.”
2032	−2.29 least satisfied	Dissatisfied	“I don't feel that the staff have been mutually working here. However, you still have to work even if you are not satisfied with it. There hasn’t been any situation when all the staff have agreed to work together with their heart.” “Actually, I don’t feel safe. While doing delivery, we don’t have anything besides gloves. We don’t have boots and masks so we have to be careful.”

Note: JSI range: −7.37 (minimum); 8.35 (maximum)

**Table 4:** Discrepant cases when comparing qualitative and quantitative job satisfaction data

ID number	Job Satisfaction Index (JSI) (quartile category)	Interpretation of qualitative interview	Maternal and Newborn Health Worker (MNHW) interview quotes
2008	−0.12 less satisfied	Satisfied	“The society over here is good. We have no problem when they come here to take service. I feel good. We have never faced problem even in night shifts. I feel good.” “I enjoy it here. Everyone has their section and their own responsibility. They work in their time and if there is any problem then we work together.” “There are delivery sets and equipment needed for Medical Abortion and check up. We have everything.”
1032	−0.65 Less satisfied	Satisfied	“People ask for me to conduct their delivery, even though it is another person’s duty. They trust me a lot and I am happy that everybody likes me. I do whatever I can. I feel confident while working and I am happy by myself.” “Nothing is easy here. There is not enough equipment, and there is no cleaner so it is difficult to clean the health facility.”
2029	−1.78 least satisfied	Satisfied	“I have an identity here. Everyone is familiar and I have made close personal friends too. After I came here, I started knowing my neighbours, sisters, and because of their love and care, it is difficult to leave this place.” “It is enjoyable when everyone does their work with mutual understanding. Because everyone is helpful and close, it is enjoyable.”
2007	−2.1 least satisfied	Satisfied	“We have access to public transport. There are *Tharu* (ethnic group) communities here…. they trust us. It is easy to work where we are trusted.” “There are many close friends of mine here. The other staff are my close friends.”

Note: JSI range: −7.37 (minimum); 8.35 (maximum)

### Quantitative validation: validity, reliability and internal consistency

This section uses survey data and accepted statistical tests described earlier, to measure the content validity, construct validity and internal consistency of the survey.

As shown in Table 5, more MNHWs were from Kailali district (43 %) and most were aged 20–29 years (44 %). Over two-thirds belonged to an upper caste group, while approximately 25 % belonged to Dalit or disadvantaged groups. MNHWs worked in a variety of posts, with most being ANMs (65 %). More MNHWs worked in health posts (47 %) and few in district or zonal hospitals (9.5 % each). Over half of the sampled MNHWs had worked in their current health facility for more than 2 years (54 %) and only 9 % had worked in their current post for less than one month. Over half of respondents (53 %) earned between 10,000 and 14,999 Nepali Rupees per month.

**Table 5:** Demographic characteristics and mean JSS of MNHWs

Variable	*n*	%	Mean job satisfaction index (JSI)	Standard deviation
*District*				
Dailekh	40	29.20	1.02	2.34
Kailali	59	43.07	−0.75	2.36
Banke	38	27.74	0.09	1.32
*Gender*				
Female	137	100	0.00^b^	2.23
*Age group*				
18–19 years	6	4.38	1.41	2.99
20–29 years	60	43.8	−0.18	2.38
30–39 years	43	31.39	−0.06	2.16
40–49 years	25	18.25	0.34	1.79
50 years or older	3	2.19	−1.05	1.36
*Ethnicity*				
Dalit Hill	4	2.92	0.3	1.6
Dalit Terai^Ɨ^	1	0.73	1.4	.
Disadvantaged Janajati Hill	14	10.22	−0.6	2.7
Disadvantaged Janajati Terai	16	11.68	−1.2	2.0
Relatively advantaged Janajati	9	6.57	1.4	2.9
Upper caste	93	67.88	0.1	2.1
*Job title*				
Auxiliary Nurse Midwife	88	64.23	−0.16	2.12
Senior Auxiliary Nurse Midwife	23	16.79	0.25	1.36
Staff Nurse	13	9.49	−0.04	4.05
Senior Staff Nurse	5	3.65	−0.44	1.40
Up-graded Maternal and Child Health Worker	8	5.84	1.44	1.63
*Type of current health facility*				
Zonal hospital	13	9.49	−0.55	2.08
District hospital	13	9.49	−0.61	4.55
Primary health centre	24	17.52	0.16	1.45
Health post	64	46.72	0.02	1.94
Sub health post	23	16.79	0.44	1.82
*Time spent in current post*				
Less than 1 month	12	8.76	2.52	2.12
1–6 months	23	16.79	−0.62	2.09
7–12 months	6	4.38	−0.55	1.57
1–2 years	22	16.06	0.02	2.07
2 years or more	74	54.01	−0.18	2.15
*Salary after tax (Nepali Rupees)* ^*a*^				
Less than 5,000	4	2.92	−1.93	0.99
5,000–9,999	39	28.47	−0.26	2.22
10,000–14,999	72	52.55	0.15	2.05
15,000–19,999	21	15.33	0.21	2.84
More than 20,000	1	0.73	2.29	.

Note: Ɨ indicates only one observation for this characteristic. As a result, the standard deviation is not calculated

^a^96.29 Nepali Eupees = 1 United States Dollar

^b^The mean value of the JSI for female is 0.000000000952

Note: JSI range: −7.37 (minimum); 8.35 (maximum)

To evaluate content validity we calculated the inter-item correlation coefficients for each dimension. For the tool to be internally consistent, the items in the tool should be moderately correlated with each other [49, 53]. Tables 6, 7, 8, 9, and 10 present the matrixes of the correlation coefficients for the five dimensions in the JSS.

**Table 6:** Inter-item correlation matrix for the pay and benefits (PB) dimension

	PB1	PB2	PB3	PB4	PB5	PB6	PB7	PB8
PB1	1							
PB2	0.49	1						
PB3	−0.03	−0.01	1					
PB4	0.21	0.22	0.14	1				
PB5	0.52	0.38	−0.03	0.26	1			
PB6	0.05	0.03	0.05	0.01	0.12	1		
PB7	0.34	0.22	−0.02	0.37	0.45	0.05	1	
PB8	0.35	0.31	0.20	0.08	0.8	0.08	0.13	1

Note: PB1 refers to item 1 in the pay and benefits dimension, PB2 to item 2 and so on

JSI range: −7.37 (minimum); 8.35 (maximum)

**Table 7:** Inter-item correlation matrix for the co-workers and supervisor (CS) dimension

	CS1	CS2	CS3	CS4	CS5	CS6	CS7
CS1	1						
CS2	0.59	1					
CS3	0.27	0.42	1				
CS4	0.43	0.53	0.37	1			
CS5	0.13	0.17	−0.02	0.30	1		
CS6	0.07	0.08	0.02	0.1	0.04	1	
CS7	0.19	0.29	−0.02	0.27	0.61	0.12	1

Note: CS1 refers to item 1 in the co-workers and supervisor dimension, CS2 to item 2 and so on

JSI range: −7.37 (minimum); 8.35 (maximum)

**Table 8:** Inter-item correlation matrix for the promotion (PR) dimension

	PR1	PR2	PR3	PR4	PR5
PR1	1				
PR2	0.14	1			
PR3	0.23	0.05	1		
PR4	0.08	0.12	0.09	1	
PR5	0.30	0.17	0.18	0.38	1

Note: PR1 refers to item 1 in the promotion dimension, PR2 to item 2 and so on

JSI range: −7.37 (minimum); 8.35 (maximum)

**Table 9:** Inter-item correlation matrix for the work environment (WE) dimension

	WE1	WE2	WE3	WE4	WE5	WE6	WE7	WE8
WE1	1							
WE2	0.19	1						
WE3	0.167	0.23	1					
WE4	0.00	0.30	0.40	1				
WE5	0.00	0.20	0.35	0.49	1			
WE6	0.11	0.21	0.31	0.15	0.12	1		
WE7	−0.01	0.23	0.25	0.12	0.26	0.21	1	
WE8	0.12	0.08	0.07	−0.00	0.10	−0.04	0.12	1

Note: WE1 refers to item 1 in the work environment dimension, WE2 to item 2 and so on

JSI range: −7.37 (minimum); 8.35 (maximum)

**Table 10:** Inter-item correlation matrix for the general satisfaction (GS) dimension

	GS1	GS2	GS3	GS4	GS5	GS6	GS7	GS8
GS1	1							
GS2	0.27	1						
GS3	0.25	0.16	1					
GS4	0.10	0.04	−0.07	1				
GS5	−0.13	0.36	−0.18	0.17	1			
GS6	0.19	0.24	0.49	−0.11	−0.04	1		
GS7	0.13	0.06	−0.04	0.09	0.06	−0.25	1	
GS8	0.02	−0.16	0.23	−0.05	−0.22	0.10	−0.04	1

Note: GS1 refers to item 1 in the general satisfaction dimension, GS2 to item 2 and so on

JSI range: −7.37 (minimum); 8.35 (maximum)

Overall, within each dimension, most items are positively and moderately correlated with each other. There are exceptions where items have low correlation coefficients or are negatively correlated. However, these low levels of correlation are not surprising given the nature of the statements. Further, these are not numerous enough to cause concern [53]. For example, in the pay and promotions dimension, the first item (*I feel that I am being paid a fair amount for the work I do*) and the third item (*Raises are too few and far between*) have a correlation coefficient of −0.03. While a negative relationship between these two items is expected, the magnitude of the coefficient is small. Perhaps this captures the feeling that few people feel that they are fairly compensated for the quantum of work that they do. In the co-workers and supervisors dimension, the fifth item (*I like the people I work with*) and the sixth item (*I find I have to work harder at my job because of the incompetence of people I work with*) have a correlation coefficient of 0.04. It is not surprising that these items are not strongly correlated, as these require the health worker to reflect on two different aspects of their interactions with their supervisors or co-workers. One may like a co-worker but need not also feel that they are competent at their job. In the work environment dimension, the first item (*Many of our rules and procedures make doing a good job difficult*) and the seventh item (*There is too much bickering and fighting at work*) have a correlation coefficient of −0.01. Again it is not surprising that these two items are not very strongly correlated. The first item captures organizational structure while the seventh item captures inter-personal relationships.

We compared responses to each item, with responses to other items in the same domain to see whether they moved in the same direction. This is true for all items in the five domains except for item PB8 in the Pay and Benefits dimension, which moved in the opposite direction. This item was dropped before constructing the JSS.

The Cronbach’s Alpha (α) coefficient of internal consistency was then used to measure the reliability of the JSI constructed from the survey data. The calculated α values for the overall JSI and for each dimension are presented in Table 11. These values are also compared to the acceptability threshold values. The tool is considered to be internally consistent if α is equal to or bigger than 0.7. Four dimensions had an α value greater than 0.65 while two dimension had α values of 0.52 and 0.42. However, the α value for the overall JSI is 0.78, which is above the 0.7 threshold value. Keeping in mind that the greater the number of items for which we calculate α, the higher the value α, these values are indicative of the internally consistency and reliability of the tool. Further, the calculated θ value was 0.82, which is higher than the overall α [52]. The results from the inter-item co-relation, comparison of responses of items within each dimension, the α and θ tests indicate that the calculated JSI is reliable and internally consistent.

**Table 11:** Cronbach's alpha (α) for the overall Job Satisfaction Index (JSI) and dimensions

Dimension	Cronbach's alpha (α)
Overall JSI	0.78
Pay and benefits	0.66
Co-workers and supervisors	0.64
Promotion	0.52
Work environment	0.65
General	0.42

The response rates for the items on the JSS were lower than those for the motivation survey administered to this sample of MNHWs [38]. Most of the MNHWs (*n* = 106, 77.4 %) did not respond to one or more items in the JSS. The Promotions dimension had the highest proportion of missing responses (8.8 %), followed by Work Environment (3.8 %), General (2.8 %), Co-workers and Supervisors (1.7 %). The Pay and Benefits dimension had the smallest proportion of missing responses (0.05 %). These results may indicate lower acceptability and/or comprehensibility of some of the questions on the JSS among respondents. The findings from the qualitative data show that these sections were the least relevant for MNHWs. Thus, it is unsurprising that response rates were lower for these dimensions. Many MNHWs were not eligible for promotion because they were on temporary contracts, or because they had not completed requisite years of experience, and many statements in the work environment dimension were irrelevant for MNHWs.

To assess construct validity and sensitivity, we explored variations in the JSI by socio-demographic and worker characteristics. The principal component calculates a JSI ranging between −7.37 and 8.35. The lower the JSI, the less satisfied the MNHW. The mean JSI was 9.52e-10 (SD = 2.23). The median JSI was 0.26. We also constructed quartiles of the JSI, that allowed us to distinguish between most satisfied, more satisfied, less satisfied and least satisfied MNHWs. Table 12 presents these quartiles with mean JSI for each quartile.

**Table 12:** Mean Job Satisfaction Index (JSI) by quartile

Quartile	Mean JSI
Least satisfied	−2.83
Less satisfied	−0.42
More satisfied	0.82
Most satisfied	2.51

Note: JSI range: −7.37 (minimum); 8.35 (maximum)

Examining the mean JSI by demographic characteristics (Table 5), we find that, on average, the highest JSIs were observed for MNHWs from Dailekh (1.02, SD 2.34), MNHWs, aged 18–19 years (1.41, SD 2.99), MNHWs belonging to the Dalit Terai ethnic group (1.4, SD -)^1^ and the relatively advantaged Janajati group (1.4, SD 2.9). We also observed highest JSIs for upgraded MCHWs (1.44, SD 1.63), MNHWs working in a sub-health post (0.44, SD 1.82) and MNHWs who had spent less than 1 month working at their current health facility (2.52, SD 2.12). Finally, MNHWs who earned 15,000 and 19,999 Nepali rupees had the highest JSIs of (0.21,SD 2.84). These MNHWs were also found to have the highest levels of motivation [35]. One MNHW who earned more than NPR 20,000 had a JSI of 2.29. The examination of the mean JSI by different demographic and socio-economic characteristics offered evidence that the index was able to differentiate between levels of job satisfaction amongst the MNHWs (Table 5).

## Discussion

In this paper we sought to establish the validity and reliability of an existing survey for the measurement of health worker satisfaction in Nepal. Qualitative and quantitative methods were used to test for construct and content validity of the tool as well as its reliability and sensitivity in the rural Nepalese context.

The qualitative validation process indicates that the dimensions of job satisfaction measured in the JSS are consistent with factors affecting job satisfaction mentioned by respondents, with one important exception: context. Community ‘fit’, is an important determinant of retention of rural health workers [54]. MNHWs’ interactions with the community, place within the community and sense of support and security, affect their ability to do their job. The importance of considering context was also illustrated when we compared qualitative data with JSS scores. We found that inconsistencies of satisfied interviews and low JSS scores were related to contextual factors not included in the standard JSS.

The findings from the qualitative data suggest that adapting the work environment dimension to the local context, and adding training and job security to the promotion and general satisfaction dimensions of the tool. In our context, red tape, or administrative barriers were not relevant for MNHWs. Another example from our context was that promotion opportunities were rare in our population. However, opportunities to participate in training sessions to improve knowledge and skills were discussed as a means for progression, and access to such opportunities positively affected MNHWs’ job satisfaction. Findings from the qualitative interviews revealed that several MNHWs felt that they were job insecure, which affected their job satisfaction. However, this was not captured in the standard JSS.

The quantitative process found that the tool was within acceptable limits in reliability and validity statistical testing, and was able to capture variation in scores between MNHWs of different socio-demographic and workplace related characteristics. There were a relatively high number of missing responses, which may indicate that some respondents found it difficult to answer some questions. It is also note worthy that MNHWs who were paid a low salary for one-two years, with no job security found it difficult to answer questions about their pay and benefits Perhaps, this was because these respondents felt that these questions may have appeared to be ‘high risk’ or ‘unacceptable’ questions to answer. These respondents had also expressed feelings of job insecurity in qualitative interviews, and this may help explain some of their reluctance to respond. Researchers may also not have adequately encouraged MNHWs to respond. To reduce the risk of this problem in future studies, we recommend that the JSS is administered by trained researchers, sensitised to the possibility that respondents will feel anxious about sharing this information and that all efforts should be made to protect respondents anonymity and privacy while responding.

We dropped one statement from our analysis from the pay and benefits section (*There are benefits we do not have which we should have*) because it was performing inconsistently and not moving in the same direction as other variables. Recordings of a random sample of seven MNHWs from two districts were analysed to explore the reasons for this inconsistency. We believe that when considering ‘benefits’ MNHWs in this context also referred to environmental factors such as electricity, water, and access to public transport. We recommend that researchers clarify the type of benefit meant when asking this question.

A known limitation of this paper is that we were unable to test for criterion validity of the JSS as there is no ‘gold standard’ against which to measure job satisfaction. In such a scenario it is recommended that one test for convergent validity, which measures the degree to which two measures that theoretically should be related are in fact related [45]. This was not possible within the constraints of this study but future work may consider using the Job Description Index developed by Bowling Green State University for this purpose. An additional limitation of this research is that, for logistical reasons, we only conducted the qualitative validation process with MNHWs who were present in the health facility. Those MNHWs experiencing very high levels of job dissatisfaction might have been absent from the health facility. Therefore, there might be a small risk of positive bias in the data collected. Further, some data may have been lost in translation in having FCHVs and nurses translate some of the interviews and respondents may not have felt that they were able to speak freely through these translators.

## Conclusion

The global crisis in human resources for health requires that researchers develop and test interventions to increase the retention and productivity of health workers, particularly in rural areas. The intention to stay or leave employment is generally difficult to measure within the short time span of a trial, and therefore predictors of intent to leave, such as job satisfaction, are required.

We have tested the validity, reliability and sensitivity of the JSS among MNHWs in rural Nepal, using qualitative and quantitative methodologies. Our findings suggest that the JSS can be used in the Nepalese setting and possibly also in other low- and middle-income country settings. However, there is some locally relevant information that the tool is not able to capture. Thus we recommend minor adaptions to reflect local contexts. The work environment dimension of the tool should be adapted to reflect particular systemic characteristics for example lack of equipment and infrastructure. The general satisfaction dimension should also capture perceptions of job security, and the promotion section should include statements regarding training. Finally, we recommend a careful translation of the tool from English into another language, so that key terms (such as benefit in our case) are clearly defined and understood by respondents and researchers alike.

Our findings indicate that the JSS is able to measure job satisfaction in our population to some extent but omitted the key factor of context. Incorporating this missing dimension and appropriately adapting the tool would create a more comprehensive and possibly more sensitive measurement tool. This would provide researchers and decision-makers, working in the public health sectors in low- and middle-income countries, with a way to measure the effect of systemic changes and policies aiming to improve health worker retention and productivity.

## Abbreviations

ANM**,** auxiliary nurse midwife**;** DHO**,** District Health Office**;** DO**,** District Health Officer**;** FCHV**,** female community health volunteers**;** FGD**,** focus group discussion**;** HDI**,** human development index**;** HFMC**,** health facility management committee**;** HP**,** health post**;** JSI**,** job satisfaction index**;** JSS**,** job satisfaction survey**;** MCHW**,** maternal child health workers**;** MNHW, maternal and newborn health workers; PHC**,** primary health care centre**;** PHN**,** public health nurse**;** VDC**,** village development committee

## Footnotes

1 Only one MNHW belonged to the Dailt Terai ethnic group; hence, no standard deviation is reported.
